# The Four Faces of Competition: The Development of the Multidimensional Competitive Orientation Inventory

**DOI:** 10.3389/fpsyg.2018.00779

**Published:** 2018-05-22

**Authors:** Gábor Orosz, István Tóth-Király, Noémi Büki, Krisztián Ivaskevics, Beáta Bőthe, Márta Fülöp

**Affiliations:** ^1^Institute of Psychology, Eötvös Loránd University, Budapest, Hungary; ^2^Institute of Cognitive Neuroscience and Psychology, Research Centre for Natural Sciences, Hungarian Academy of Sciences, Budapest, Hungary; ^3^Doctoral School of Psychology, Eötvös Loránd University, Budapest, Hungary; ^4^Department of Criminal Psychology, Institute of Behavioral Sciences, Faculty of Law Enforcement, National University of Public Service, Budapest, Hungary

**Keywords:** competition, competition avoidance, exploratory structural equation modeling (ESEM), hypercompetitive orientation, measurement invariance, Multidimensional Competitive Orientation Inventory (MCOI), self-developmental competitive orientation

## Abstract

To date, no short scale exists with established factor structure that can assess individual differences in competition. The aim of the present study was to uncover and operationalize the facets of competitive orientations with theoretical underpinning and strong psychometric properties. A total of 2676 respondents were recruited for four studies. The items were constructed based on qualitative research in different cultural contexts. A combined method of exploratory structural equation modeling (ESEM) and confirmatory factor analysis (CFA) was employed. ESEM resulted in a four-factor structure of the competitive orientations and this structure was supported by a series of CFAs on different comprehensive samples. The Multidimensional Competitive Orientation Inventory (MCOI) included 12 items and four factors: hypercompetitive orientation, self-developmental competitive orientation, anxiety-driven competition avoidance, and lack of interest toward competition. Strong gender invariance was established. The four facets of competition have differentiated relationship patterns with adaptive and maladaptive personality and motivational constructs. The MCOI can assess the adaptive and maladaptive facets of competitive orientations with a short, reliable, valid and theoretically underlined multidimensional measure.

## Introduction

Research on individual differences in competitive orientations is mostly based on a unidimensional concept of competition (Ryckman et al., 1990, 1996, 2009). From the theoretical perspectives, a scale integrating the different facets of competitive orientations allows the examination of the blend of competitive attitudes which are not mutually exclusive. This measure also allows to examine simultaneously the constructive and destructive aspects of competitive orientations that can have intrapersonal and interpersonal consequences. From an applied perspective, a brief multifaceted measure can effectively assess the role of competitive orientations in a variety of settings including education, health, organizations, and sport. To our best knowledge, however, no prior measure with established factor structure could capture the multifaceted complexity of individual differences in competition. In the present research, we propose a new measure, the Multidimensional Competitive Orientation Inventory (MCOI) that could complete this shortcoming of the competition research literature.

Individual differences in competitive orientations can have critical effects on performance in achievement situations (Deutsch, 1949a; Kristof, 1996) and they can play an important role in intrapersonal and interpersonal psychological processes. As an example, for intrapersonal processes, individuals lower in trait competitiveness have decreased job dedication which results in lower performance in competitive climate (Fletcher et al., 2008). This link between trait competitiveness and high performance is also mediated by different achievement goals (Murayama and Elliot, 2012). As for interpersonal processes, a positive link was identified between peer competitiveness and body dissatisfaction among female students (Ferguson et al., 2014). Among male students, relationship was uncovered between competitiveness, self-disclosure, and closeness of friendship (Busse and Birk, 1993). In the case of both same and opposite sex friends, friendship competitiveness was linked to conflict (Singleton and Vacca, 2007). More recently, Brewer et al. (2014) found that friendship competitiveness predicted the accuracy of self-disclosure among same-sex friendship dyad. In sum, individual differences in competitive orientation influence several aspects of everyday life such as job dedication, job and task performance, body satisfaction, or closeness, conflicts, and self-disclosure in friendships.

Until the 1990s, the major ruling paradigm in competition research conceptualized competitive orientation as a unidimensional construct (Fülöp, 2009; Fülöp and Orosz, 2015). This approach defined competition as a desire to win in interpersonal situations (Helmreich et al., 1980). Additionally, it was identified as a polar opposite of cooperation (Deutsch, 1949a) which usually has detrimental effects such as aggression, hostility among the competing parties (Kohn, 1986) or ill-health (Rosenman et al., 1964).

However, in the last three decades, there has been a paradigm change regarding competitive attitudes and orientations in the field of personality psychology (Ryckman et al., 1990, 1996, 2009), social psychology (Tassi and Schneider, 1997; Schneider et al., 2011; Fülöp and Orosz, 2015), and evolutionary psychology (Charlesworth, 1996; Hawley, 2010). This line of research differentiated the unidimensional concept of competitive attitudes, orientations and strategies by identifying its different facets. Based on this approach, it can be argued that competitive orientation is a multidimensional construct that can incorporate both beneficial and detrimental aspects of social behavior.

Similarly to Tassi and Schneider (1997), some of the prior theories differentiate two main forms of competitiveness, referring to them as (a) perspectives of competitiveness (Griffin-Pierson, 1990) or (b) facets of competitiveness (Kayhan, 2003). In these differentiations, one of the dimensions is related to the strong desire of winning, dominating or being superior to others, while the other refers to the desire for excellence, obtaining a goal, bringing out the best one can do, mastering the task, and developing oneself.

Apart from the distinction of Griffin-Pierson (1990) and Kayhan (2003), a different classification has also been suggested with two independent dimensions of competitiveness (Houston et al., 2002; Harris and Houston, 2010) which differ from the above-mentioned ones based on content. The first one is *enjoyment of competition* which refers to personal attitudes toward one’s competitive behavior (e.g., liking, enjoying/disliking, avoiding competition), whereas the second dimension is *contentiousness* which refers to attitudes toward avoidant behavior in arguments and conflicts.

Complementing and further developing the existing models with two dimensions, Franken and Brown (1995) distinguished five dimensions of competitiveness: satisfaction that comes from improving one’s performance; satisfaction of performing well; motivation to put forth effort; reference for difficult tasks, and desire to win, referring to the importance of winning over others. Later, Franken and Prpich (1996) described new dimensions that are related to the negative consequences of evaluations. One of the negative consequences is called as *self-image concerns* and it refers to disliking competition because of the fear of failing or being perceived negatively. The second is called as *performance concerns*, referring to disliking competitive situations because other people are inclined to have high expectations for the self. The third dimension, called *distraction of attention*, directly refers to the detrimental effects of evaluation on attention. Franken and Brown (1995) and Franken and Prpich (1996) provided the empirical bases for the multidimensional competitiveness scales. However, their measures did not have impeccable psychometric properties: both the factor structure and the reliability of the scales showed serious limitations.

Another typology was created by Ryckman et al. (1990, 1994, 1996, 2009), Ryckman and Hamel (1992). In a both complex and comprehensive approach, Ryckman et al. (1990) did not define competitiveness as a personality trait, but rather as a set of attitudes or orientations. Therefore, they differentiated three independent competitive attitudes: personal-development competitive attitude, hypercompetitive attitude, and competition avoidance. However, to our best knowledge, no prior study examined the construct validity of these measures in terms of factor structure either separately or together. One of the rationale of the present study is to use recent factor analytic approach by building on Ryckman et al.’s work (Ryckman et al., 1990, 1994, 1996, 2009) and assess simultaneously these and one additional competitive orientation with a brief, comprehensive measure with good validity and reliability. For this reason, below we detail Ryckman et al.’s three competitive attitudes.

Regarding the *personal-development competitive attitude* (PDCA), the primary focus is on personal growth and on the enjoyment and mastery of the task in a competitive situation. The goal attainment and competition outcome (i.e., on winning) is important, but not at the expense of the derogation of other competitors (Ryckman et al., 1996). Individuals with dominant PDCA are motivated by self-achievement, strive for doing their best and to improve and discover themselves during the process of competition (Ryckman and Hamel, 1992). PDCA is positively associated with higher self-esteem, task enjoyment, self-development, self-discovery, achievement, affiliation, and other indicators of social and psychological health, whereas it is negatively related to derogating others, neuroticism, excessive exhibitionism, aggression and dominance (Ryckman et al., 1996, 1997; Burckle et al., 1999; Thornton et al., 2009, 2013; Collier et al., 2010; Hibbard and Buhrmester, 2010; Mudrack et al., 2012).

Ryckman et al. (1990) based their investigations of *hypercompetitive attitude* (HCA) on Horney’s (1937) hyper-competitiveness concept. According to her theory, hyper-competitiveness is an exaggerated, neurotic form of competitiveness. Individuals scoring high on the HCA scale have a very strong need to compete and to win at any cost, because their self-worth is based on competition, thus they feel more powerful by winning a competition and, at the same time, winning makes them feel superior to others. They perceive their competitors as enemies and they are not afraid to use unfair strategies in order to win or derogate other competitors. HCA is found to be mostly related to maladaptive outcomes. This orientation is associated with low self-esteem, low self-actualization, low optimal psychological health, high neuroticism, high aggression, high dominance, and high exhibition scores; higher mistrust, Machiavellianism, dogmatism, and narcissism (Ryckman et al., 1990, 1994, 1996, 2009).

Research on the third orientation type, *competition avoidance*, was also based on Horney’s (1937) work. Competition avoidance is another major form of neurotic competitive orientation that refers to *“the need of individuals to check their ruthless ambition because of excessive fear of losing the affection and approval of others as a consequence of either being successful in competition with others or through failure in such competition”* (Ryckman et al., 2009, p. 176). Such individuals are stressed as success or failure in competition would elicit rejection and dislike from others, thus they try hard to avoid competing and proving their competence in achievement situations. Beyond fearing success, they also very stressed because of potential failures. They fear the others’ denigration toward them and they usually feel embarrassed or humiliated by competitive defeat. Higher competition avoidance correlates with higher neuroticism and lower optimal psychological health. Competition avoiders lack confidence in themselves in achievement situations by engaging in self-handicapping behavior (Ryckman et al., 2009).

Considering the complexity of individual differences in competitive orientations, it is not surprising that it has been assessed and operationalized in diverse ways. Most of the research dealt with competitiveness or competitive orientation either as simply a negative attitude or as a construct with an approaching and avoidant aspect. However, it is possible to be indifferent and be non-interested in competition as well, which means no approach and no avoidance of competitive situations, but being non-interested and non-motivated into either direction. This aspect has never been directly measured previously as a separate orientation, just indirectly based on low scores in the existing scales. If one focuses on only one facet, it is possible to overlook the relative importance of other aspects. For instance, if one has high level of self-developmental competitive orientation and low levels of all of the other competitive facets provides a very different pattern compared to the cases when one has high level of self-developmental orientation and high level of lack of interest toward competition at the same time. The first refers to a dominantly learning-focused competitive orientation, whereas the second refers to an orientation when the individual is basically not interested in competitive situations but if this person occasionally steps into the competition she/he focuses on the self-development. Therefore, measuring only one facet of competitive attitudes without considering simultaneously the other dimensions can be misleading. Besides the unidimensional measures (Ryckman et al., 1990, 1996, 2009) there were attempts to create multidimensional ones (Griffin-Pierson, 1990; Franken and Brown, 1995; Franken and Prpich, 1996; Kayhan, 2003; Newby and Klein, 2014). However, the psychometric properties of these questionnaires do not perfectly meet the most recent requirements and standards. The majority of these studies did not use the factor analytic approach (Ryckman et al., 1990, 1996, 2009) or if they did so the factor structure and the reliability of the scales showed more or less serious limitations (Franken and Brown, 1995; Franken and Prpich, 1996; Newby and Klein, 2014). Furthermore, none of the pre-existing measures had good factor structure on the one hand and could assess the multidimensional nature of competitive orientations on the other hand. The present multidimensional measure aims to overcome these shortcomings by providing a short, reliable, and valid measure with strong psychometric properties that can assess simultaneously different facets of competitive attitudes.

For these reasons, the aim of the present research was the construction of a comprehensive scale which (a) can measure the most important facets of competition, (b) which can be applied to one’s competitive orientation in different fields (i.e., being as context-free as possible), (c) which is short and thus can be applied in combination with a battery of other instruments, and (d) which has strong psychometric properties in terms of factor structure, validity and reliability.

Therefore, we constructed the MCOI by generating items on the basis of previous qualitative studies. Then, in four studies, the inventory was validated by using a construct validity approach that includes both within-network and between-network validity analyses (Shavelson et al., 1976; Marsh et al., 2005a). Within-network validity refers to the features (e.g., structure or components) of the construct and whether it reflects the collected data. The model fit of the data (i.e., whether the hypothesized model fits the data) was assessed with different samples of elementary, high school, university and comprehensive samples using both exploratory and confirmatory factor analysis (CFA) in Studies 1 and 2, respectively. By employing different samples for all the analyses, cross-validation also becomes possible, giving further support for the model. These analyses were complemented with gender invariance testing in Study 3. When within-network validity is adequately established, one can proceed with the between-network validity by examining the relationships between the factors of the scale and other related constructs. This form of validity was assessed in Study 4 by examining the correlations between the MCOI and other personality and motivational measures.

## Study 1 – the Development of the Multidimensional Competitive Orientation Inventory (MCOI)

In the first study, our goal was to develop a pool of items that correspond to each of the competition facets described in the “Introduction” section. These items were created based on previous interviews and responses to open-ended questionnaire carried out by Fülöp (1992a,b, 1995, 1999, 2004, 2009), Fülöp and Orosz (2006); Schneider et al. (2011). After generating an initial set of items, a short version was created by reducing the number of items (For details see section Method and Results) so that the final short version can be administered in larger questionnaire batteries. Subsequently, exploratory analyses were performed to investigate its factor structure and psychometric properties which was followed by a CFA for the purpose of cross-validation.

### Method

#### Item Construction

In several studies with high school students, college students, teachers, business people of different nationalities open-ended questionnaires requiring free descriptive answers and semi-structured interviews were applied in order to reveal the qualitatively different concepts of competition of the respondents (Fülöp, 1992a,b, 1995, 1999, 2004; Fülöp and Orosz, 2006; Schneider et al., 2011). Participants were also asked about their personal attitude toward competition. The statements related to personal competitiveness were collected and categorized. The categorization was based on phenomenography (Marton, 1986; Fülöp, 2004) which aims to identify qualitatively different ways of perceiving and understanding of a phenomenon. Based on these studies, with the phenomenographic method, the authors sorted perceptions of competition into specific categories. Four categories of competitive orientation emerged that provided the phenomenographic essence of competitive orientations: hypercompetitive orientation, self-developmental competitive orientation, anxiety-driven competition avoidant orientation, and lack of interest toward competition. In order to create an initial item-set of these qualitatively different factors, we used the exact words of the respondents participated in the aforementioned previous qualitative studies. The main aim was to create items which were (1) concise and easy to understand; (2) clearly belonged to a given dimension, but not to other ones; (3) were not double-barreled; and (4) were not suggestive. To minimize potential group decision making biases, an iterative approach was applied with multiple sessions: in the first session, an initial number of 28 items were created by the authors. In a second session, these items were reviewed and revised in a group meeting where a professor of competition and 18 MA Psychology students were present. Eight items were dropped from this initial pool as a result of redundancy, lack of clarity, or the misfit between the item and its respective factor. This resulted in a final 20 items for the subsequent analysis with a six-point response option (1 = Not true to me at all; 6 = Completely true to me). For details of further item selection see section “Results”.

#### Procedure

In the present study, two different samples were used for the purpose of validation and cross-validation. For Sample 1, the item set was tested on a sample of 525 university students (female = 46.77%), aged between 18 and 92 (*M* = 30.51, *SD* = 0.69). Sample 2 was representative in terms of gender, age, level of education among those Hungarians who used Internet at least once a week. As the data gathering was online, this sample was not representative among those who do not use internet at least once a week. For this reason, we use the term of comprehensive sample for these sorts of samples. The participants were selected randomly from an Internet-enabled panel including 88000 members with the help of a Hungarian publisher company in July 2015. For the preparation of this sample, a multiple-step, proportionally stratified, probabilistic sampling method was employed. Individuals were removed from the panel if they gave responses too quickly (i.e., without paying attention to their response) and/or had fake (unused) e-mail addresses.

This final comprehensive sample of 500 participants had the following characteristics: gender (female = 251), age (*M* = 35.05 years; *SD* = 11.97 years, ranging from 15 to 59 years), education (20.0% had primary level of education, 22.8% had vocational school degree, 38.2% graduated from high school and 19% had higher education degree), and place of residence (20.2% in capital city, 20.1% in county capitals, 34.6% in cities and 25.2% in villages).

In both cases, data collection was conducted in accordance with the Helsinki Declaration and was approved by the United Ethical Review Committee for Research in Psychology (EPKEB). Participants were informed about the content of the questionnaire. They volunteered for the study, they did not receive any kind of compensation for the participation.

#### Statistical Analysis

For the statistical analyses, SPSS 22 and Mplus 7.3 (Muthén and Muthén, 1998–2015) were used. The initial number of 20 items were examined on the basis of three criteria which were already established in previous studies (e.g., Fahlman et al., 2013; Bőthe et al., 2018): (1) corrected item-total correlations, (2) normality in terms of skewness and kurtosis (Muthén and Kaplan, 1985; Curran et al., 1996), and (3) content validity (Haynes et al., 1995) compared to the other items and competition in general.

In the next part, exploratory structural equation modeling (ESEM; Marsh et al., 2009; Morin et al., 2013) was conducted. This analytic approach is a synergy between exploratory factor analysis and CFA in the sense that it allows the explicit expression of item-level cross-loadings, while also making it possible to target these cross-loadings to zero (Tóth-Király et al., 2018). While the items were heavily structured to be related to the main factors, completely pure items measuring only one construct are rarely achieved, resulting in a certain degree of overlap (i.e., true score association) with the other related factors (Asparouhov et al., 2015) which is often the case in psychological studies (e.g., Morin and Maïano, 2011; Perera, 2015; Litalien et al., 2017; Tóth-Király et al., 2017a,c). As these cross-loadings could be indicative of imperfect items that might need further modification, we opted to carry out ESEM analyses, where the cross-loadings are expressed but targeted to be as close to zero as possible (Browne, 2001) to more closely approximate the general specification of CFA. Subsequently, we re-examined the MCOI on the comprehensive sample with CFA to test whether the factor structure holds with the more restrictive approach (i.e., cross-loadings are forced to zero).

Following common guidelines (Brown, 2015), multiple goodness of fit indices were taken into consideration (Hu and Bentler, 1999; Marsh et al., 2005b) when evaluating a model: the Comparative Fit Index (CFI; ≥0.95 good, ≥0.90 acceptable), the Tucker–Lewis Index (TLI; ≥0.95 good, ≥0.90 acceptable), the Root-Mean-Square Error of Approximation (RMSEA; ≤0.06 good, ≤0.08 acceptable) with its 90% confidence interval and the test of close fit (CFit; ≥0.10 good, ≥0.05 acceptable), and the Standardized Root Mean Square Residual (SRMR; ≤0.05 for good, ≤0.10 for acceptable). The Akaike Information Criterion (AIC) was also observed in order to compare the different models with lower values indicating better model fit. Finally, the AIC values were transformed to Akaike weights (i.e., conditional probabilities) to facilitate the interpretation of the results (Wagenmakers and Farrell, 2004).

Internal consistency was measured with Cronbach’s alpha (Nunnally, 1978) with its acceptable (0.70) and good (0.80) threshold values. However, as this indicator can be less reliable (Sijtsma, 2009; Rodriguez et al., 2016), two further reliability indices were observed. First, factor determinacy (FD), ranging from 0 to 1, which describes the correlations between the true and estimated factor scores with values closer to one indicating higher levels of reliability (Muthén and Muthén, 1998–2015). Second, composite reliability was also calculated by following the formula of Raykov (1997). It might be considered as a more reliable indicator as it accounts for both the factor loadings and their respective measurement errors. A general rule of thumb is to have a value higher than 0.60 for acceptable and higher than 0.70 for good reliability (Bagozzi and Yi, 1988; Hair et al., 2014).

### Results

In the first part of the analysis, each of the 20 items were examined on the basis of (1) corrected item-total correlations, (2) normality in terms of skewness and kurtosis, and (3) content validity compared to the other items and competition in general (see **Table 1**). By following these criteria, a total of eight items were eliminated, resulting in 12 items that were retained for the subsequent analyses. Skewness and kurtosis scores were under ±2 (Curran et al., 1996), therefore no items were excluded for this reason. However, six items were eliminated as a result of their relatively lower item-total correlations (LIC4, LIC 5, HCA4, HCA5, SDCA4, and SDCA5) and two items because of their content validity (in terms of final content-based refinement) compared to the other items (ADCA4, ADCA5). These items were selected prior to the ESEM and CFA.

**Table 1:** Initial item set of the Multidimensional Competitive Orientation Inventory.

Items	IIC	Skew.	Kurt.
I rarely feel motivated to compete with somebody. (LIC1)	**0.67**	**0.13**	**-1.07**
There is always something I’d rather do than taking part in a competitive situation. (LIC2)	**0.66**	**0.31**	**-1.02**
I don’t care about competitions. (LIC3)	**0.72**	**0.67**	**-0.72**
I avoid competition because rather I enjoy life. (LIC4)	0.63	0.53	-0.68
I choose rather amusement instead of competition. (LIC5)	0.63	0.11	-0.91
The most important is winning, no matter what. (HCO1)	**0.71**	**1.11**	**0.33**
I am willing to do whatever it takes to win. (HCO2)	**0.78**	**1.19**	**0.49**
I will do anything to win, even nasty things. (HCO3)	**0.65**	**0.95**	**0.11**
For the sake of winning I can be even aggressive. (HCO4)	0.65	0.83	-0.38
If I really want to win in a competitive situation the end justifies the means. (HCO5)	0.63	0.74	-0.58
I feel distressed in a competitive environment, so I avoid them whenever I can. (ADCA1)	**0.81**	**0.83**	**-0.30**
I feel pressured in competitive situations. (ADCA2)	**0.81**	**0.57**	**-0.86**
Even the smallest competition makes me feel anxious. (ADCA3)	**0.70**	**0.73**	**-0.51**
I feel uncomfortable in competitive situations. (ADCA4)	0.80	0.58	-0.78
I can’t stand stress deriving from competition. (ADCA5)	0.79	0.54	-0.76
Competitive situations allow me to bring the best out of myself. (SDCO1)	**0.77**	**-0.43**	**-0.63**
I enjoy testing myself in competitive situations. (SDCO2)	**0.80**	**-0.33**	**-0.71**
I enjoy competition as it allows me to discover my abilities. (SDCO3)	**0.81**	**-0.58**	**-0.52**
Competition helps me to experience what I’m able to do compared to others. (SDCO4)	0.72	-0.73	0.21
I like comparing my knowledge to others’. (SDCO5)	0.72	-0.42	-0.42

*LIC, lack of interest in competition; HCO, hypercompetitive orientation; ADCA, anxiety-driven competition avoidance; SDCO, self-developmental competitive orientation; IIC, Inter-item Correlation; skew., skewness; kurt., kurtosis. Bold letters represent the final items.*

Next, ESEM was performed to investigate the factor structure of the remaining items. This solution yielded adequate fit to the data (χ^2^ = 21.931, df = 24, *p* = 0.583; CFI = 1; TLI = 1; RMSEA = 0.000 [90% CI 0.000 -0.032]; CFit = 0.999; SRMR = 0.007, AIC = 19300.628)^1^. The results indicate strongly target loadings (|λ| = 0.56–0.91, *M* = 0.77) and minimal cross-loadings (|λ| = 0.00–0.20, *M* = 0.06). The latent inter-factor correlations, reliability indices and descriptive statistics can be seen in **Table 2**.

**Table 2:** Results of the exploratory structural equation modeling on the Multidimensional Competitive Orientation Inventory items.

	Multidimensional Competitive Orientation Inventory factors			
	Lack of interest toward competition	Hyper-competitive	Anxiety-driven competition avoidant	Self-developmental competitive
(1) I rarely feel motivated to compete with somebody.	**0.79**	*-0.04*	*-0.02*	*0.00*
(2) There is always something I’d rather do than taking part in a competitive situation.	**0.62**	*-0.06*	0.20	*0.06*
(3) I don’t care about competitions.	**0.72**	*0.02*	*-0.04*	-0.17
(4) The most important is winning, no matter what.	*0.10*	**0.87**	*0.01*	*0.06*
(5) I am willing to do whatever it takes to win.	*-0.01*	**0.91**	*-0.02*	*-0.05*
(6) I will do anything to win, even nasty things.	-0.16	**0.56**	*0.03*	*0.00*
(7) I feel distressed in a competitive environment, so I avoid them whenever I can.	0.12	*0.03*	**0.77**	*-0.03*
(8) I feel pressured in competitive situations.	*0.05*	*-0.01*	**0.75**	*-0.08*
(9) Even the smallest competition makes me feel anxious.	-0.11	*-0.03*	**0.85**	*0.03*
(10) Competitive situations allow me to bring the best out of myself.	*-0.01*	*0.04*	0.07	**0.80**
(11) I enjoy testing myself in competitive situations.	-0.20	*0.00*	*-0.04*	**0.67**
(12) I enjoy competition as it allows me to discover my abilities.	0.12	*-0.01*	-0.09	**0.89**

	**Inter-factor correlations**				

Lack of interest toward competition	–			
Hyper-competitive orientation	-0.37	–		
Anxiety-driven competition avoidance	0.60	-0.21	–	
Self-developmental competitive orientation	-0.77	0.31	-0.63	–

	**Reliability indices**				

Cronbach’s alpha	0.82	0.82	0.86	0.86
Factor determinacy	0.93	0.94	0.93	0.94
Composite reliability	0.75	0.83	0.83	0.83

	**Descriptive statistics**				

Mean (SD) [observed range: 1–6]	3.02 (1.36)	2.18 (1.15)	2.62 (1.34)	4.02 (1.26)
Skewness (SD)	0.39 (0.11)	1.05 (0.11)	0.69 (0.11)	-0.44 (0.11)
Kurtosis (SD)	-0.78 (0.21)	0.52 (0.21)	-0.45 (0.21)	-0.44 (0.21)

*All factor loadings are standardized. Loadings in bold represent the final items and all are statistically significant at p < 0.001. Non-significant cross-loadings are in italics.*

Similarly to the ESEM model, the first-order CFA model indicated good fit to the data in the comprehensive sample (χ^2^ = 120.665, df = 48, *p* < 0.001, CFI = 0.979; TLI = 0.971; RMSEA = 0.055 [90% CI 0.043 -0.067]; CFit = 0.237; SRMR = 0.035). This solution confirmed that the scale has appropriate factor structure.

#### Labels of the Factors

In sum, results suggest that the four-factor solution is the most adequate compared reflecting on the four facets of competition. The first three facets are directly rooted in the theory of Horney (1937). The first facet—hypercompetitive orientation—is closely related to Ryckman et al.’s (1990) hypercompetitive attitude dimension. However, in the present case hypercompetitive orientation appears in terms of a very strong result orientation in which the end justifies the means. The second facet—self-developmental competitive orientation—is closely related to the personal-developmental competitive attitudes of Ryckman et al. (1996). However, in the present case the focus is on the self, and ability improvement and this factor does not directly relate to preserving good relationship with rivals. The third factor—anxiety-driven competition avoidance—is related to Ryckman et al.’s (2009) competition avoidance. However, in the present case competition avoidance was driven by a rather general anxiety deriving from the process of competition and it is not explicitly driven from the fear of losing the approval of affection of others. The fourth facet is—lack of interest in competition—is different from the previously described ones as it is related to the disinterest in competitive situations. It does not explicitly represent approach or avoidant motivations, but the lack of motivation regarding competitive situations. In the following, we intended to test the adequacy of the proposed factor structure using CFA with a comprehensive sample and to cross-validate it on both elementary and high school samples.

## Study 2 – Confirming and Cross-Validating the Factor Structure of the MCOI

The goal of Study 2 was to confirm the factor structure of the MCOI and also to compare it to the previous ESEM and CFA results on a separate comprehensive sample. After examining the final solution, the secondary goal was to cross-validate it on more specific samples of elementary and high school students.

### Methods

#### Procedure

In the present study, three different samples were used for the purpose of validation and cross-validation. For the comprehensive Sample 1, the same procedure was applied as in Study 1 and was recruited via the same online methods. In the case of Sample 2 and 3, paper-pencil tests were administered. Participants of all three samples were informed about the content of the questionnaire. They volunteered for the study, they did not receive any kind of compensation for the participation. Regarding pupils and high school students, the schools and parents were also informed through an opt-out passive consent. They filled out the questionnaire during class and they were encouraged to give remarks and raise questions.

#### Participants

##### Sample 1

This comprehensive sample (*N* = 900) consisted of 445 male and 455 female respondents (*M*_age_ = 36.687 years; *SD*_age_ = 14.29 years; age range = 14–93 years). Regarding the highest completed level of education, 16.2% (*n* = 146) of the respondents have primary level of education, 23.9% (*n* = 215) have vocational school degree, 37.5% (*n* = 338) graduated from high school, and 21.2% (*n* = 190) have higher education degree. Regarding the place of residence 21% (*n* = 189) of the respondents live in the capital, 20.2% (*n* = 182) live in the county towns, 34.3% (*n* = 309) live in towns and 24.5% (*n* = 220) live in villages.

##### Sample 2

This sample of elementary school students consisted of 216 participants (female = 116, male = 90, 10 did not indicate) from four elementary schools, aged between 12 and 15 years (*M* = 13.49; *SD* = 0.70). One school was located in a Hungarian village (*n* = 50, 23%), one was in a town (*n* = 94, 43.3%), one was in a county town (*n* = 46, 21.2%), and one was in the capital (*n* = 24, 11.1%) of Hungary. Two pupils did not report the location of their school.

##### Sample 3

The high school student sample (*N* = 192) consisted of 80 female and 104 male students (eight of them did not respond this question) from a Hungarian high school, aged between 14 and 20 years (*M* = 16.44; *SD* = 1.24). The students were living in a town (*n* = 80, 43.5%) or in a village (*n* = 102, 55.4%).

### Results

**FIGURE 1:**
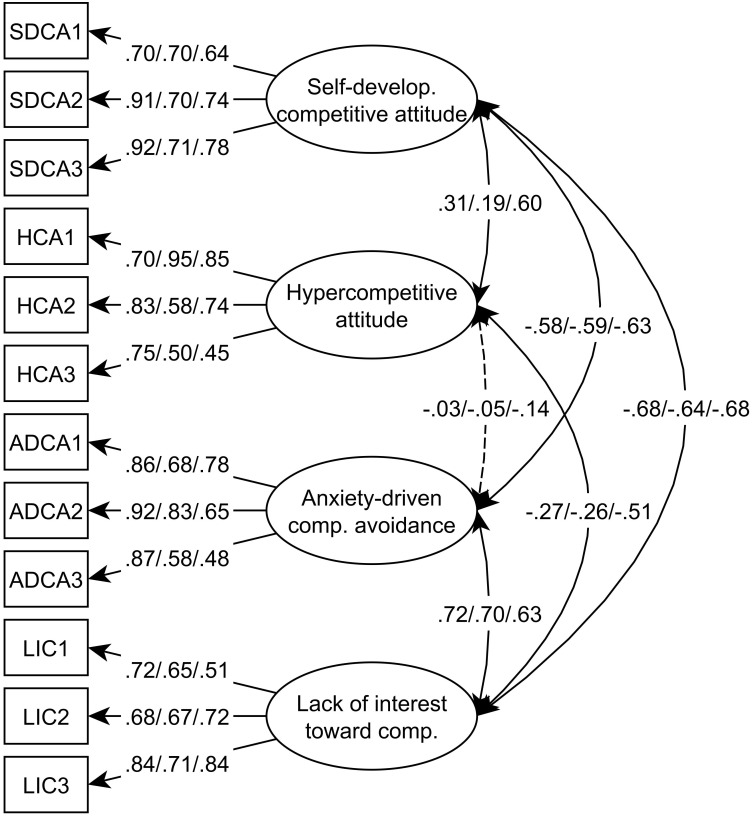
The final confirmatory factor analysis model of the Multidimensional Competitive Orientation Inventory. One-headed arrows represent standardized factor loadings; two-headed arrows represent correlations, and dashed arrows represent non-significant paths. Regarding factor loadings and correlations, the first number refers to Sample 1, the second number refers to Sample 2, and the third number refers to Sample 3.

Similarly to the results of Study 1, the first-order model indicated good fit to the data on this second comprehensive sample (χ^2^ = 116.539, df = 48, *p* < 0.001; CFI = 0.990; TLI = 0.987; RMSEA = 0.040 [90% CI 0.031 -0.049]; CFit = 0.965; SRMR = 0.024, AIC = 29477.086). This solution corroborates our findings. In the next step, the results were cross-validated on the high school and elementary school samples which also demonstrated good fit to the data: elementary school sample (χ^2^ = 70.676, df = 48, *p* < 0.050; CFI = 0.967; TLI = 0.955; RMSEA = 0.047 [90% CI 0.020 -0.069]; CFit = 0.565; SRMR = 0.046, AIC = 7744.744), high school sample (χ^2^ = 67.734, df = 48, *p* < 0.050; CFI = 0.970; TLI = 0.959; RMSEA = 0.047 [90% CI 0.015 -0.072]; CFit = 0.545; SRMR = 0.046, AIC = 7395.783)^2^. Standardized factor loadings and inter-factor correlations can be seen in **Figure 1**.

All internal consistency and reliability indices (Cronbach’s alpha, factor determinacy, and composite reliability) demonstrated good values (see **Table 3**), giving further strong support for the validity and reliability of the MCOI.

**Table 3:** Reliability indices and descriptive statistics of the Multidimensional Competitive Orientation Inventory.

Scales	Range	Sample 1 (*N* = 900)					Sample 2 (*N* = 216)					Sample 3 (*N* = 192)				
		α	CR	FD	*M*	*SD*	α	CR	FD	*M*	*SD*	α	CR	FD	*M*	*SD*
(1) LIC	1–6	0.81	0.83	0.94	3.19	1.25	0.71	0.72	0.88	2.51	1.08	0.74	0.74	0.91	3.33	1.31
(2) HCO	1–6	0.84	0.85	0.93	2.42	1.15	0.70	0.73	0.95	2.57	1.07	0.70	0.73	0.91	2.93	1.17
(3) ADCA	1–6	0.92	0.93	0.97	2.59	1.21	0.75	0.74	0.90	2.11	1.00	0.68	0.68	0.88	2.39	1.09
(4) SDCO	1–6	0.86	0.89	0.96	4.17	1.20	0.74	0.75	0.88	4.89	0.94	0.76	0.78	0.91	4.12	1.20

*LIC, lack of interest in competition factor; HCO, hypercompetitive attitude; ADCA, anxiety-driven competition avoidance; SDCO, self-developmental competitive attitude; α, Cronbach’s alpha value; CR, composite reliability; FD, factor determinacy; M, mean; SD, standard deviation.*

## Study 3 - Measurement Invariance Testing of the MCOI

Is the factor structure of the MCOI similar for men and women? Can the results be generalized to different subgroups? Between-group comparisons are important psychometric aspects of an instrument such as the MCOI. If the factor structure could be replicated across different samples, then any further comparisons are meaningful and thus can be generalized. This can be done by performing invariance testing (Meredith, 1993; Vandenberg and Lance, 2000; Vandenberg, 2002).

Multiple levels of invariance testing can be distinguished: first, *configural* model where all groups had the same factor structure and all parameters are freely estimated; second, *weak* (metric) model where factor loadings were constrained to be equal across the groups; third, *strong* (scalar) model where intercepts are set to be equal; fourth, *strict* (residual) model where error variances needed to be equal. Moreover, latent *variances-covariances* and *latent factor means* could also be investigated. Therefore, the following goal of this study was to test gender invariance on the MCOI.

### Method

#### Participants

For the purposes of measurement invariance testing, three adult samples were combined from previous samples: the university sample of 525 participants from Study 1, the comprehensive sample of 500 participants from Study 1 and the comprehensive sample of 900 participants from Study 2. For further sample characteristics, see previous studies.

#### Statistical Analysis

For measurement invariance, baseline models were identified for the different subsamples. Next, parameter constraints were gradually imposed from the least restrictive to the most restrictive one (e.g., Meredith and Teresi, 2006; Morin et al., 2013; Tóth-Király et al., 2017b): configural invariance, weak invariance, strong invariance, strict invariance, invariance of the variance-covariance matrix, and latent mean invariance. In model evaluation, the same goodness-of-fit indices and their respective cut-off values were applied as in previous studies. When comparing the different models, relative differences of fit indices were calculated (Cheung and Rensvold, 2002; Chen, 2007): ΔCFI ≤ 0.010; ΔTLI ≤ 0.010; and ΔRMSEA ≤ 0.015. Specifically, parsimony-corrected indicators (such as TLI and RMSEA) might have great importance (Marsh et al., 2009; Morin et al., 2013).

### Results

Goodness-of-fit indices for the estimated models can be seen in **Table 4**. First, configural model with no parameters constrained to equality in the two groups was estimated. This model provided adequate fit to the data (CFI = 0.985; TLI = 0.979; RMSEA = 0.047 [90% CI 0.041 -0.053]; CFit = 0.795). All subsequent models (weak, scalar, residual, latent variance-covariance and latent means) were successfully estimated with the gradually imposed constraints. While most χ^2^ and Δχ^2^ test were significant, other model fit indices (ΔCFI ≤ 0.010; ΔTLI ≤ 0.010; ΔRMSEA ≤ 0.015) did not decrease more than the recommended cut-off values. In many cases, parsimony-corrected fit indicators (i.e., TLI and RMSEA) even improved with the addition of invariance constraints. In sum, the present results provided strong support for the conclusion for the gender invariance of the MCOI on the level of latent means.

**Table 4:** Goodness-of-fit statistics and information criteria for the estimated models on the Multidimensional Competitive Orientation Inventory.

Gender invariance										
**Model**	**χ^2^ (df)**	**CFI**	**TLI**	**RMSEA**	**90% CI**	**CFit**	**Comparison**	**Δχ^2^ (df)**	**ΔCFI**	**ΔTLI**	**ΔRMSEA**

MGa. Male	134.205^∗^ (48)	0.983	0.976	0.029	0.039–0.059	0.536	–	–	–	–	–
MGb. Female	165.040^∗^ (48)	0.986	0.981	0.045	0.038–0.053	0.833	–	–	–	–	–
MG1. Configural	299.245^∗^ (96)	0.985	0.979	0.047	0.041–0.053	0.795	–	–	–	–	–
MG2. Weak	305.616^∗^ (104)	0.985	0.981	0.045	0.039–0.051	0.923	MG2–MG1	6.371 (8)	0.000	0.002	-0.002
MG3. Strong	332.529^∗^ (112)	0.984	0.981	0.045	0.040–0.051	0.917	MG3–MG2	26.913^∗^ (8)	-0.001	0.000	0.000
MG4. Strict	357.956^∗^ (124)	0.983	0.981	0.044	0.039–0.050	0.960	MG4–MG3	25.427 (12)	-0.001	0.000	-0.001
MG5. Latent variance-covariance	406.102^∗^ (134)	0.980	0.980	0.046	0.041–0.051	0.902	MG5–MG4	48.146^∗^ (10)	-0.003	-0.001	0.002
**MG6. Latent means**	**498.073^∗^ (138)**	**0.973**	**0.974**	**0.052**	**0.047–0.057**	**0.238**	**MG6–MG5**	**91.971^∗^ (4)**	-**0.007**	-**0.006**	**0.006**

*CFA, confirmatory factor analysis; χ^2^, Chi-square; df, degrees of freedom; CFI, comparative fit index; TLI, Tucker–Lewis Index; RMSEA, root-mean-square error of approximation; 90% CI, 90% confidence interval of the RMSEA; CFit, RMSEA’s test of close fit; Δχ^2^, Chi-square difference test; ΔCFI, change in CFI value compared to the preceding model; ΔTLI, change in the TLI value compared to the preceding model; ΔRMSEA, change in the RMSEA value compared to the preceding model; Bold letters indicate the final models and final levels of invariance that were achieved.; ^∗^p < 0.01.*

## Study 4 - Assessing the Convergent Validity of the MCOI

Previous studies of this research demonstrated that the MCOI had strong within-network validity (Shavelson et al., 1976; Marsh et al., 2005a) in terms of factor structure, internal consistency, reliability and high levels of gender invariance. The aim of this study was to investigate the between-network validity (Shavelson et al., 1976; Marsh et al., 2005a) of the MCOI. For this purpose, we examined the correlations of the different factors of MCOI with motivational and personality constructs.

In each of the analyses, the links between the factors of MCOI and other related variables were measured such as resiliency, positivity, perfectionism, and achievement motivations in terms of work versus family orientation. The hypothesized correlations were based on theory and previous research. We expected that *self-developmental competitive orientation* would be positively related to personality traits as perceived resilience as an indicator of psychological health (Ryckman et al., 1996; Burckle et al., 1999); positivity in terms of self-esteem, optimism and life-satisfaction (Ryckman et al., 1996; Burckle et al., 1999); positive aspects of perfectionism (Ryckman et al., 1996; Thornton et al., 2011), and all mastery, work-related and competitive aspects of achievement motivation (Ryckman et al., 1996, 1997; Hibbard and Buhrmester, 2010). Therefore, we expected that SDA has generally positive pattern of correlations. In contrast to the self-developmental competitive orientation, *anxiety-driven competition avoidance* would have a generally negative correlational pattern. Namely, despite this is one of the least investigated aspects of competitive orientation, on the basis of Ryckman et al.’s (2009), we expected that anxiety-driven competition avoidance would be negatively related to personality traits as perceived resilience, positivity in terms of self-esteem, optimism and life-satisfaction, positive aspects of perfectionism, and all mastery, work-related and competitive aspects of achievement motivation. In the case of *hypercompetitive orientation*, on the basis of prior work of Ryckman et al. (1990, 1994, 1996, 2009), we expected that it is related to the negative aspects of perfectionism, and competitive forms of achievement motivations. With regards to *lack of interest competition avoidance*, we expected similar correlational pattern as in the case of anxiety-driven competition avoidance, with smaller correlational coefficients except for competitive motivations and perfectionism because this competitive orientation is fundamentally against both competitive or perfectionist striving.

### Method

#### Participants

A total of 343 participants were recruited for the study from high schools and universities based on a call distributed by teachers. The participants filled in an online questionnaire in the educational institutions. The age of the participants ranged from 13 to 57 (*M*_age_ = 19.48, *SD*_age_ = 5.09) with 214 females and 129 males. From participants under the age of 18 (*n* = 130), parental consent was obtained prior to the beginning of data collection. With regards to their highest level of education, 188 participants (54.8%) completed primary education, 85 (24.8%) had secondary education, and 66 (19.2%) completed college/university level education. 4 participants (1.2%) did not specify their level of education. 53 participants (15.5%) lived in capital city, 78 (22.7%) lived in county capitals, 96 (28%) lived in cities, 57 (16.6%) lived in villages and 59 (17.2%) lived abroad.

#### Measures

##### Connor-Davidson Resilience Scale (CD-RISC)

We used the 10-item version of the CD-RISC (Campbell-Sills and Stein, 2007) that assesses individuals’ perceptions of their resilience. The authors of the CD-RISC broadly defined resilience as “*personal qualities that enable one to thrive in the face of adversity*” (Connor and Davidson, 2003, p. 76). The items comprising the CD-RISC-10 assessed individuals’ perceptions of their abilities to adapt to change, deal with unexpected events, cope with illness and injury, handle unpleasant feelings, maintain positivity in the face of stress, and cope with obstacles (Campbell-Sills and Stein, 2007). Respondents rated items on a scale from 0 (not true at all) to 4 (true nearly all the time).

##### Positivity

The Positivity Scale is a short measurement of positive orientation—in terms of optimism, life satisfaction, and self-esteem—developed by Caprara et al. (2012). The scale is composed of eight items (e.g., “I’m satisfied with my life” or “I have great faith in the future”). Answers are provided on a five-point scale ranging from 1 “Strongly disagree” to 5 “Strongly agree.”

##### Almost Perfect Scale-Revised (APS-R)

The APS-R (Slaney et al., 2001) is designed to measure the positive and negative aspects of perfectionism. The scale contains 23 self-report items with two subscales: High Standards (7 items; e.g., *“I expect the best from myself.”*), and Discrepancy (12 items; e.g., *“My best just never seems to be good enough for me.”*). Similarly to Rice et al. (2014), order items were not included in the current analysis. Respondents provided responses on a 5-point scale ranging from 1 (strongly disagree) to 5 (strongly agree).

##### Consequences of perfectionism (COPS)

The COPS (Kim, 2010; Stoeber et al., 2013) was used to capture consequences of perfectionism. The scale contains 10 items, with six items measuring positive consequences (e.g., “*Perfectionism drives me to be motivated*.”) and four items measuring negative consequences (e.g., “*Perfectionism hinders me from staying on track in my performance.*”). Participants provided their responses on a five-point scale ranging from 1 (extremely untrue of me) to 5 (extremely true of me).

##### Work and Family Orientation Questionnaire (WOFO)

Work and Family Orientation Questionnaire (Spence and Helmreich, 1983) is a 19-item scale intended to measure different aspects of achievement motivation. The scale contains three subscales: Work Orientation (6 items, e.g., “*There is satisfaction in a job well done.*”), Mastery (e.g., “*I prefer to work in situations that require a high level of skill.*”), and Competitiveness (e.g., “*I enjoy working in situations involving competitions with others*”). Responses are rated on a 5-point scale ranging from 1 (strongly disagree) to 5 (strongly agree).

### Results

Descriptive statistics and internal consistency indices are presented in **Table 5**, while the associations between the different competitive orientations and adaptive-maladaptive outcomes are presented in **Table 6**. Partial correlations were performed to control for the covariance between the different facets: when conducting the partial correlations between the competitive factors (e.g., hypercompetitive orientation) and the outcomes, all other competitive factors (i.e., lack of interest, anxiety-driven and self-developmental) are controlled for. Generally, most associations were in line with the expectations. Self-developmental competitive orientation had a positive association with resilience, positivity, the discrepancy factor of perfectionism, the positive consequences of perfectionism, and all achievement motivations. Hypercompetitive orientation was only associated with the high standards factor of perfectionism, the negative consequences of perfectionism and the work-related achievement motivation. Although the lack of interest and anxiety-driven competitive factors had high associations in the previous studies of this investigation, they are rather distinct on the basis of their associations with the correlates. More specifically, while the lack of interest factor had a small positive correlation with resilience, it was negatively related to the discrepancy factor of perfectionism and the work-related achievement motivation. On the other hand, while the anxiety-driven factor was inversely related to resilience and the discrepancy factor of perfectionism (negatively and positively, respectively), it was also positively related to the high standard factor of perfectionism and the negative consequences of perfectionism.

**Table 5:** Descriptive statistics and reliability indices for Study 4.

Scales	Range	Mean (*SD*)	Cronbach’s α
(1) MCOI lack of interest in competition	1–6	2.62 (1.07)	0.77
(2) MCOI hypercompetitive orientation	1–6	2.50 (1.14)	0.83
(3) MCOI anxiety-drive competition avoidance	1–6	2.24 (1.06)	0.83
(4) MCOI self-developmental competitive orientation	1–6	4.96 (0.93)	0.83
(5) CD-RISC	1–5	3.82 (0.59)	0.83
(6). Positivity	1–5	3.70 (0.61)	0.80
(7) APS-R discrepancy	1–5	2.69 (0.88)	0.92
(8) APS-R high standards	1–5	4.14 (0.59)	0.79
(9) COPS positive	1–5	4.20 (0.56)	0.82
(10) COPS negative	1–5	1.85 (0.71)	0.83
(11) WOFO mastery	1–5	3.45 (0.53)	0.63
(12) WOFO competition	1–5	3.49 (0.76)	0.78
(13) WOFO work	1–5	4.25 (0.46)	0.63

*SD, standard deviation; MCOI, Multidimensional Competitive Orientation Inventory; CD-RISC, Connor-Davidson Resilience Scale; APS-R, Almost Perfect Scale-Revised; COPS, Consequences of Perfectionism; WOFO, Work and Family Orientation Questionnaire.*

**Table 6:** Partial correlations of the Multidimensional Competitive Orientation Inventory with adaptive and maladaptive personality and motivational constructs.

Scales	LIC		HCO		ADCA		SDCO	
	*r*	95% CI	*r*	95% CI	*r*	95% CI	*r*	95% CI
(1) CD-RISC	0.12*	0.015, 0.223	-0.04	-0.145, 0.066	-0.23**	-0.327, -0.128	0.24**	0.138, 0.337
(2) Positivity	0.05	-0.056, 0.155	-0.02	-0.125, 0.086	-0.11	-0.213, -0.005	0.17**	0.066, 0.271
(3) APS discrepancy	-0.15**	-0.251, -0.045	0.07	-0.036, 0.174	0.21**	0.107, 0.309	0.22**	0.117, 0.318
(4) APS high standards	-0.02	-0.125, 0.086	0.14*	0.035, 0.242	0.29**	0.190, 0.384	0.07	-0.036, 0.174
(5) COPS positive	0.02	-0.086, 0.125	0.00	-0.105, 0.105	0.09	-0.016, 0.194	0.35**	0.254, 0.439
(6) COPS negative	-0.06	-0.164, 0.046	0.19**	0.086, 0.290	0.20**	0.097, 0.299	-0.09	-0.194, 0.016
(7) WOFO mastery	-0.04	-0.145, 0.066	-0.05	-0.155, 0.056	0.02	-0.086, 0.125	0.26**	0.159, 0.356
(8) WOFO competition	-0.01	-0.115, 0.095	-0.02	-0.086, 0.125	0.03	-0.076, 0.135	0.17**	0.066, 0.271
(9) WOFO work	-0.34**	-0.430, -0.243	0.37**	0.275, 0.457	0.08	-0.026, 0.184	0.18**	0.076, 0.280

*LIC, lack of interest in competition factor; HCO, hypercompetitive orientation; ADCA, anxiety-driven competition avoidance; SDCO, self-developmental competitive orientation; APS Discrepancy, Almost Perfect Scale Discrepancy; APS Standards, Almost Perfect Scale High Standards; COPS Positive, Positive Consequences of Perfectionism; COPS Negative, Negative Consequences of Perfectionism; WOFO Mastery, Work and Family Orientation Mastery; WOFO Competition, Work and Family Orientation Competition; WOFO Work, Work and Family Orientation Work; ^∗^p < 0.05, ^∗∗^p < 0.01.*

## General Discussion

Individuals can perceive competition in qualitatively different ways. Walt Disney said once that *“I have been up against tough competition all my life. I wouldn’t know how to get along without it.”* which is fundamentally different from Béla Bartók’s view on competition *“Competitions are for horses, not artists.”* These quotations refleca dominant competition orientation (e.g., hypercompetitive or avoidant orientation), these orientations can co-exist. Therefore, our goal was to build upon unidimensional competition measures (Ryckman et al., 1990, 1996, 2009) and construct a multidimensional measure that—in contrast to previous ones (Griffin-Pierson, 1990; Franken and Brown, 1995; Franken and Prpich, 1996; Kayhan, 2003)—can meet the most recent psychometric requirements and standards. According to the results, we were able to achieve this goal by creating a short multidimensional measure with strong within- and between-network validity (Shavelson et al., 1976; Marsh et al., 2005a).

In terms of within-network validity, with multiple studies and separate comprehensive samples, thorough and exhaustive statistical analyses (both ESEM and CFA) were performed to investigate the factor structure of the MCOI. All analyses suggested that MCOI has an adequate factor structure. These results were corroborated by other reliability and validity indices. The Cronbach’s alpha, the composite reliability and factor determinacy of the four factors also underscored that MCOI is a valid and reliable measure. To our best knowledge, no prior measure focusing on different aspects of competitive orientations was examined by invariance testing. In the case of MCOI, gender invariance on the level of latent means was achieved, giving strong support for the generalizability of the MCOI across male-female subgroups. In sum, on the basis of these results MCOI is a brief measure that, has good factor structure, with reliable subscales and high level of gender invariance.

The MCOI model is compatible with the theoretical background of previous multidimensional models. However, compared to the models of Franken and Brown (1995) or later, Franken and Prpich (1996), the present model met contemporary psychometric standards. Furthermore, Frankel and Brown’s measure did not include one of the important dimensions of individual differences in competitive orientations: competition avoidance. Later, Franken and Prpich (1996) complemented this omission. However, their measure only focused on avoidance aspects related to the outcome of competition, but not on the process of competition in terms of anxiety-driven competition avoidance. Besides building on other unidimensional measures of competition, the present model is based on phenomenographical qualitative research (Fülöp, 1992a,b, 1995, 1999, 2004, 2009; Fülöp and Orosz, 2006; Schneider et al., 2011) as well by including hypercompetitive, personal-developmental, and competition-avoidant orientations of Ryckman et al. (1990, 1996, 2009).

The MCOI can distinguish competition approach, competition avoidant and competition neutral orientations. It includes two competition approach orientations: hypercompetitive and self-developmental competitive attitudes. The main difference between these two is that the hypercompetitive orientation is strongly result-oriented, while the self-developmental competitive orientation is strongly process-oriented. In the MCOI model, the competition avoidance is anxiety-driven. Finally, the neutral dimension is related to the interest vs. uninterest toward competitive situations. Therefore, this measure can assess both approach and avoidant aspects of competitive orientations, it can differentiate between the result- and process-orientation, and it can assess the general interest toward competitive situations.

In terms of between-network validity, we examined whether the factors of the scale were associated with other factors in expected directions. Self-developmental competitive orientation was positively related to hypercompetitive orientation and negatively related to anxiety-driven and uninterested competitive orientations. This might be explained along the approach vs. avoidance dimensions. The positive link between self-developmental and hypercompetitive orientations can be explained by that both belong to the competitive approach dimension. The negative links between self-developmental orientation vs. competitive avoidant and uninterested facets can be attributed to the notion that these latter orientations express an avoidant or neutral standpoint regarding competition. Hypercompetitive orientation was negatively or non-significantly related to anxiety-driven competition avoidance and lack of interest toward competition. The negative correlations can be explained by the approach vs. avoidance and neutral distinction. However, in this case we might suppose that hypercompetitive orientation can appear more easily simultaneously with anxiety-driven competitive orientation. More specifically, considering both the relatively strong negative relationship between self-developmental competitive orientation and anxiety-driven competitive orientation and the weak or non-significant link between hypercompetitive anxiety-driven competitive orientation, the probability of being simultaneously hypercompetitive and being anxiety-driven competition avoidant can appear as more probable than being self-developmental competitive and anxiety-driven competition avoidant at the same time. Future studies should explore this supposition with person-centered methods. Finally, it is important to mention that the anxiety-driven and uninterested orientations were positively related. This link is also reasonable, as both of these represent disengagement regarding competition, but for different reasons. This can also be the subject of further research.

The focus of the *Self-developmental competitive orientation* is the self and ability improvement. In line with prior studies (Ryckman et al., 1996, 1997; Burckle et al., 1999; Hibbard and Buhrmester, 2010; Thornton et al., 2011) and our expectations, it was positively related to the personality factors of resilience (i.e., personal qualities that enable one to thrive despite adversities) and positivity (i.e., positive orientation in terms of optimism, life satisfaction, and self-esteem), positive aspects of perfectionism, and diverse forms of achievement motivations. Somewhat unexpectedly, it was also related to the Almost Perfect Scale’s discrepancy measure. One possible explanation could be that self-development is a continuous process with seemingly no end, hence the desired perfect state cannot truly be reached. Furthermore, it was also related to perfectionism that leads to positive outcomes regarding motivations and performance; achievement motivation in terms of accomplishing work with satisfaction regardless the competitive nature of the situation and in terms of mastery and challenge seeking. In sum, self-developmental competitive orientation is related to diverse personality and motivational adaptive behaviors. This competitive orientation is related to beneficial psychological processes that promote positive aspects of mental health, perfectionism and learning.

*Hypercompetitive orientation* is characterized by very strong result orientation (i.e., winning) in competitive situations in which the end may justify the means. On the basis of prior work of Ryckman et al. (1990, 1994, 1996, 2009), we expected that it would be related to the negative aspects of perfectionism, and competitive forms of achievement motivations. In line with our expectations, this competitive orientation was weakly but positively related to negative aspects of perfectionism and it was also associated with the work-related achievement motivation, indicating that hypercompetitive individuals desire and prefer to work hard. Furthermore, dissimilarly to self-developmental competitive orientation, hypercompetitive orientation was unrelated to resilience, and positivity as personality characteristics. However, it was weakly related to high perfectionistic standards. In sum, the simultaneous presence of competitive achievement motivation and perfectionism stemming from high standards reflect on the result-oriented aspect of hypercompetitive orientation.

*Anxiety-driven competition avoidance orientation* focuses on the general anxiety deriving from the process of competition. We expected that it would show an inverse negative correlational pattern compared to self-developmental competitive orientation. The results partially supported our expectations: anxiety-driven individuals who avoid competition might be less resilient, have higher standards in perfectionism and have a higher discrepancy between their perfectionist ideals and their actual state. Interestingly, this avoidant behavior is also associated with negative consequences of perfectionism. In sum, anxiety-driven competitive orientation, similarly to Ryckman et al. (2009) has a generally maladaptive correlational pattern in terms of high-standard-oriented perfectionism coupled with lower level of resilience.

*Lack of interest in competitive orientation* is related to the disinterest in competitive situations and the lack of any approach or avoidance motivation regarding competitive situations. Concerning this facet, we expected similar correlational pattern as in the case of anxiety-driven competition avoidance, but with smaller correlational coefficients. The results were not in line with our expectations: this factor was weakly related to resilience and perfectionism discrepancy, while moderately and negatively related to work-related achievement motivation. Indeed, when individuals are not interested in competitions (regardless of it being overt or hidden), they are less likely to put additional effort in their work. Individuals characterized by this orientation may be less concerned with expectations of others in competitive or other achievement situations in which a “well-done job” is required.

To summarize, these convergent validity results suggest that competition avoidance and the lack of interest toward competition do not appear to have strong adaptive personality and motivational links. Their correlates showed a slightly less adaptive correlational pattern than in the case of hypercompetitive orientation. In contrast, self-developmental competitive orientation had a generally positive personality and motivational correlational pattern. These results can have both theoretical and practical implications.

### Theoretical Implications

Prior self-reported assessments of individual differences in competition had shortcomings in terms of not using the factor-analytic approach (Ryckman et al., 1990, 1996, 2009) or if they did so the factor structure and the reliability of the scales showed more or less serious limitations (Franken and Brown, 1995; Franken and Prpich, 1996; Newby and Klein, 2014). The present multidimensional measure aimed to overcome these shortcomings and meet the current psychometric standards by providing a short, reliable, and valid measure with strong psychometric properties that can assess simultaneously different facets of competitive orientations.

From the broader theoretical perspective, it might be important to consider the potential positive aspects of competition and competitive orientations. The present results regarding self-developmental competitive orientation fit a broader research stream emphasizing potential positive aspects of competition in terms of performance, interpersonal relationships, resource control, intrinsic and extrinsic motivations (Hurlock, 1927; Sims, 1928; Reeve et al., 1985; Bornstein et al., 1990; Wentzel, 1991; Epstein and Harackiewicz, 1992; Erev et al., 1993; Young et al., 1993; Reeve and Deci, 1996; Ryckman et al., 1996; Tassi and Schneider, 1997; Harackiewicz et al., 1998; Fülöp, 1999, 2001, 2004; Hawley, 2003, 2006; Tjosvold et al., 2003, 2006; Tauer and Harackiewicz, 2004). The present study along with prior ones regarding personal-developmental competitive orientation demonstrate that such orientation can be beneficial in many field of life as resilience, positivity, positive aspects of perfectionism, psychological health, task enjoyment, achievement, self-development, self-discovery, autonomy, exciting life, financial success, desire for success, individual self-esteem, and ethical idealism (Ryckman and Hamel, 1992; Ryckman et al., 1996, 1997; Collier et al., 2010). Despite, according to previous meta-analyses, reviews, and impactful studies, competition was predominantly perceived as a negative and harmful phenomenon leading to negative outcomes on performance, problem solving, personal relationships, and lower intrinsic motivation (Lewis, 1944; Lewis and Franklin, 1944; Deutsch, 1949a,b; Johnson and Johnson, 1974, 1979, 1982; Deci et al., 1981; Johnson et al., 1981; Vallerand et al., 1986; Qin et al., 1995). However, in these investigations, it would have been important to consider the participants’ orientation toward competition, instead of being forced to step in situations with competitive goal settings without considering and measuring their competitive orientations.

Further research should explore whether individuals with dominantly self-developmental orientation behave similarly to individuals with other dominant competitive patterns. It would be especially important as in a relatively recent meta-analysis, Murayama and Elliot (2012) found that trait competitiveness had positive effect on performance when performance-approach goals were present, but it was negatively related to performance when performance avoidance goals mediated this link. Self-developmental competitive attitudes—hand in hand with mastery-approach goals—might be investigated in future experimental studies. On the basis of the correlational pattern one might expect that these competitive orientations can be related to various adaptive outcomes.

On the basis of prior studies and the present correlational pattern, we can expect less adaptive personality and motivational outcomes from the strongly result-oriented, competition-approach hypercompetitive orientation (compared to the self-developmental competitive orientation). However, considering the overall correlational pattern, lack of interest in competition and especially anxiety-driven competition avoidant orientation shows even less adaptive personality and motivational profile. These patterns lead to the most important practical implication.

### Practical Implications

Competitive orientation refers to one’s thoughts, emotions, and anticipated or actual behaviors regarding competitive situations. On the basis of the first two studies, this perception of one’s competitive orientation can be diverse. On the basis of Study 4, this diversity is related to fundamentally different personal and motivational profiles. Institutions as schools or workplaces can shape these orientations and if these institutions create norms in which competitiveness in itself should be diminished, adaptive self-developmental competitive orientation will be less likely to be reinforced. At the same time, the lack of interest toward competition and anxiety-driven competition avoidance can be reinforced. Therefore, based on the results of the present multidimensional measure, it might be more beneficial to create such norms that promote mastery-based, learning-, and process-oriented self-developmental competitive orientation instead of eliminating every form of it. In sum, it would be better to create constructive competitive institutional norms (Tjosvold et al., 2003, 2006; Fülöp and Takács, 2013; Orosz et al., 2013a,b) that can foster dominantly self-developmental competitive orientation.

### Limitations and Future Directions

Although the present study has several strengths (such as the application of comprehensive samples as well as diverse analytic procedures), it is not without limitations. All measures were completed on self-report scales; therefore, they could lead to possible biases (e.g., recall bias). In future studies, these scales should be complemented with objective tools that could directly measure not only self-reported competitive behavior while, at the same time, respecting the privacy of the individuals. Despite convergent validity was established, the present study did not focus on assessing discriminant validity of the MCOI. Future research is needed to overcome this shortcoming. As correlational design was used in the present research, causality cannot be inferred. Moreover, within the framework of the current research, only cross-sectional studies were conducted which did not allow the examination of different life events that might influence competitive orientations. A longitudinal design would be beneficial in examining how potential life events (e.g., new workplace, unemployment) could have an impact on the individual’s competitive attitudes. Furthermore, from both theoretical and practical perspectives, it would be beneficial to examine the adaptive and maladaptive blend of competitive orientations. Replications in other cultures could also be performed as different cultural and economic characteristics might also influence one’s competitive orientations. Regarding the MCOI itself, further examination is needed to assess its temporal stability as well as its convergent, divergent and predictive validity.

## Conclusion

On the basis of four studies, we could identify four main aspects of competitive orientations: hypercompetitive, self-developmental, anxiety-driven competition avoidant, and lack of interest toward competition. High levels of gender invariance were established. The Multidimensional Competitive Orientations Inventory is short, and grasps the multifaceted nature of individual differences in competition. For the first time, this measure can allow the identification of adaptive and maladaptive blends of competitive attitudes.

## Author Contributions

GO and MF contributed to the study design, literature review, data gathering, manuscript writing, and to the data analyses and interpretation. IT-K and BB contributed to data analyses and interpretation, and to the manuscript writing. NB and KI contributed to the literature review, data analyses, and to the manuscript writing. All authors commented on the draft and contributed to the final version, approved the publication of the manuscript, and agreed to be accountable for all aspects of the work.

## Conflict of Interest Statement

The authors declare that the research was conducted in the absence of any commercial or financial relationships that could be construed as a potential conflict of interest.

## Footnotes

1 The fact that the CFI and the TLI equal one could be attributed to multiple reasons: in this four-factor model, the chi-square value is smaller than the degrees of freedom, and as the TLI is calculated from these two, it is greater than one such as in the present case. However, TLI (or non-normed fit index) is non-normed, meaning that its values could sometimes fall outside the range of 0–1 (Schermelleh-Engel et al., 2003). Also, high sampling variability can also result in TLI values higher than 1 (Marsh et al., 1996). RMSEA as well as TLI incorporate a penalty for the lack of parsimony which can result in improved fit (Marsh, 2007). It is possible that TLI “over-compensated” for the more parsimonious model. As for CFI, it is capped at one which is its upper threshold.

2 Upon reviewer’s request we tested the fit of the three-factor model (CFI = 0.935; TLI = 0.916; RMSEA = 0.100 [90% CI 0.093 -0.108]; CFit = 0.054, AIC = 29868.030) but was worse compared to the four-factor one as apparent by the fit indices. It was true if we apply the well-established guidelines for model comparisons (Cheung and Rensvold, 2002; Chen, 2007): for CFI and TLI, an increase of at least 0.010 would indicate a significantly better model; for RMSEA, a decrease of 0.015 would indicate a significantly better model. Furthermore, the difference was also salient if we use χ2 comparison (396.944, df = 3, *p* < 0.001) or AIC weight comparison between the three- and four factor models based on the method of Wagenmakers and Farrell (2004).
